# Association between body composition, sarcopenia and pulmonary function in chronic obstructive pulmonary disease

**DOI:** 10.1186/s12890-022-01907-1

**Published:** 2022-03-26

**Authors:** Nathalie Martínez-Luna, Arturo Orea-Tejeda, Dulce González-Islas, Laura Flores-Cisneros, Candace Keirns-Davis, Rocío Sánchez-Santillán, Ilse Pérez-García, Yael Gastelum-Ayala, Valeria Martínez-Vázquez, Óscar Martínez-Reyna

**Affiliations:** 1 Heart Failure and Respiratory Distress Clinic, Cardiology Service, Instituto Nacional de Enfermedades Respiratorias “Ismael Cosío Villegas”, Calzada de Tlalpan 4502 Col Sec XVI CP, 14080 Del Tlalpan, Mexico City, Mexico; 2 Department of Clinical Research, Instituto Nacional de Cancerología, Mexico City, Mexico

**Keywords:** Chronic obstructive pulmonary disease, Pulmonary function, Sarcopenia, Body composition, Skeletal muscle mass

## Abstract

**Background:**

Chronic Obstructive Pulmonary Disease (COPD) is characterized by progressive and irreversible airflow limitation. Different factors that modify pulmonary function include age, sex, muscular strength, and a history of exposure to toxic agents. However, the impact of body composition compartments and sarcopenia on pulmonary function is not well-established. This study aimed to evaluate how body composition compartments and sarcopenia affect pulmonary function in COPD patients.

**Methods:**

In a cross-sectional study, patients with a confirmed diagnosis of COPD, > 40 years old, and forced expiratory volume in the first second /forced vital capacity ratio (FEV_1_/FVC) < 0.70 post-bronchodilator were included. Patients with cancer, HIV, and asthma were excluded. Body composition was measured with bioelectrical impedance. Sarcopenia was defined according to EWGSOP2, and pulmonary function was assessed by spirometry.

**Results:**

185 patients were studied. The mean age was 72.20 ± 8.39 years; 55.14% were men. A linear regression adjusted model showed associations between body mass index, fat-free mass, skeletal muscle mass index, appendicular skeletal muscle mass index, and phase angle (PhA), and sarcopenia with FEV_1_ (%). As regards FVC (%), PhA and exercise tolerance had positive associations.

**Conclusion:**

Body composition, especially PhA, SMMI, ASMMI, and sarcopenia, has a significant impact on pulmonary function. Early detection of disturbances of these indexes enables the early application of such therapeutic strategies in COPD patients.

## Introduction

Chronic Obstructive Pulmonary Disease (COPD) is a treatable and avoidable illness characterized by persistent respiratory symptoms and progressive and irreversible airflow limitation [1]. According to the World Health Organization, COPD is considered a public health problem because globally it is the third leading cause of death [2].

Pulmonary function is modified by different factors, including age, sex, weeks of gestation, muscular strength, the immune system, and a history of exposure to toxic agents such as tobacco, wood smoke, and asbestos [3–5].

The pulmonary function can be estimated by spirometry. Spirometry reveals pulmonary dynamic volumes: forced expiratory volume in the first second (FEV_1_), forced vital capacity (FVC), and the ratio of forced expiratory volume in the first second to forced vital capacity (FEV_1_/FVC). Several studies have demonstrated that FEV_1_ reduction is a significant predictor of mortality in the general population [6, 7] and a marker of cardiovascular mortality [8]. Therefore, it is necessary to know which factors affect it.

Patients with COPD have alterations in the body composition compartments of fat-free mass (FFM), appendicular skeletal muscle mass index (ASMMI), skeletal muscle mass index (SMMI), fat mass (FM), phase angle (PhA), and muscular function. Loss of muscle mass and muscular function have multifactorial origins. The factors involved include oxidative stress, hypoxia, disuse, malnutrition, a higher catabolic state, and glucocorticoid use [9]. The prevalence of muscle wasting ranges from 15 to 40% in patients with COPD [10, 11]. Weight and muscle mass loss are associated with diminished muscle strength, walking speed, exercise tolerance, pulmonary alterations, and worse prognosis in COPD patients [10, 12–14].

Previous studies in other populations have shown that reduced skeletal muscle mass is associated with lower pulmonary function [15, 16]. Moreover, the strength of the respiratory muscles impacts pulmonary function [17]. In COPD patients, upper limb muscle strength has been positively associated with the FEV_1_/FVC ratio and respiratory muscle strength [5]. However, the role of the fat-free mass index (FFMI) is unclear. Maddocks et al. found no difference in predicted FEV_1_% between subjects with low and normal FFMI. On the other hand, Machado et al. showed that subjects with low FFMI had lower predicted FEV_1_% [18]. No evidence has been reported about the impact of SMMI or ASMMI on pulmonary function.

Sarcopenia is a progressive and generalized skeletal muscle disorder characterized by low muscle strength and low muscle mass that can lead to falls, fractures, physical disability, and mortality [10, 19]. The loss of muscle mass and sarcopenia have multifactorial origins, including mitochondrial abnormalities, diminished protein synthesis, diminished intake of essential amino acids for protein synthesis, hypoxemia which interferes with protein synthesis, and increased proteolysis due to a pro-inflammatory state. In addition, glucocorticoid use, which promotes proteolysis and acidosis, is common in COPD patients. Combined with these factors, physical inactivity promotes muscle atrophy [20]. A meta-analysis performed by Benz et al. estimates that the prevalence of sarcopenia in COPD patients is 21.6% (95% CI; 14.6 to 30.9%) [21]. In COPD, sarcopenia patients have lower FEV_1_ than patients without sarcopenia [22].

Phase angle (PhA) is an important body composition variable of bioelectrical impedance analysis (BIA). A low PhA suggests lower cellularity, membrane integrity, cellular function, malnutrition, status, impaired quality of life, and worse prognosis [14, 22, 23]. In COPD patients, this manifests as reduced muscle strength [14, 22] Maddocks et al. showed that COPD patients with a PhA below of fifth percentile of age, sex and BMI-stratified reference values had low predicted FEV_1_% and lower physical activity than COPD patients with normal PhA [13].

However, the association between body composition compartments and sarcopenia with pulmonary function in COPD patients is not well-established. Thus, the objective of this study was to evaluate how body composition compartments and sarcopenia may affect pulmonary function in COPD patients.

## Materials and methods

A cross-sectional study was performed. The data were obtained during outpatient evaluations carried out during routine consultations of patients of Mexican origin with COPD between August 1, 2019, and March 31, 2020.

Patients with a confirmed diagnosis of COPD according to the Global Initiative for Chronic Obstructive Lung Disease (GOLD) recommendations [24] were included. The subjects were > 40 years old, and spirometry with a post-bronchodilator FEV_1_/FVC ratio < 0.70. Patients with diagnoses of cancer, HIV, and asthma were excluded.

### Outcome measures

Body composition, anthropometry, pulmonary function, clinical and demographic variables were evaluated as part of the clinical management provided to the patients who came to the Institute.

#### Anthropometry

Weight and height were measured according to the manual reference of anthropometric standardization [25]. All subjects wore light clothing and were barefoot. Body mass index (BMI) was calculated by dividing the total body weight (kilograms) by the height in meters squared.

#### Body composition

Body composition and phase angle (PhA) were measured with whole-body bioelectrical impedance using a four-pole mono-frequency equipment RJL Quantum IV analyzer (RJL Systems®, Clinton Township, MI, USA). Phase angle was calculated using the equation: arctan (Reactance/Resistance) x (180º /π) using the PhA Software (RJL Systems®). The standard technique [26] was used. The measurements were all performed by the same operator, in the morning, in a comfortable area, free of drafts, and with portable electric heaters. The subjects were fasting and should not have exercised eight hours before or consumed alcohol 12 h before the study. During the entire study, the person was supine with arms separated from the trunk at about 30º and legs separated at about 45º.

The area was cleaned with alcohol, and electrodes were placed on the hand and ipsilateral foot. Resistance and reactance were registered, and PhA. Fat-free mass (FFM) and fat mass were estimated by RJL Systems’ software BC 4.2.2. Appendicular skeletal muscle mass index (ASMMI) was assessed according to Sergi’s formula (27): ASMMI (Kg/m^2^) = [-3.964 + (0.227*(Height^2^ (cm)/Resistance) + (0.095*Weight) + (1.384*Sex) + (0.064*Reactance) /Height (m^2^)].

Skeletal muscle mass index (SMMI) was assessed according Janssen’s formula (28): SMM (kg) = [(Height^2^ cm/Resistance × 401) + (gender × 3.825) + (age x − 0.071) + 5.102], and SMMI (kg/m^2^) = SMMI /Height (m^2^).

#### Handgrip strength

Handgrip strength was measured with a mechanical Smedley Hand Dynamometer (Stoelting, Wood Dale, UK) according to the technique described in Rodriguez et al. [29].

#### Sarcopenia

Sarcopenia was defined according to EWGSOP2 [19] in men as ASMMI < 7 kg/m^2^ and handgrip strength < 27 kg and in women as ASMMI < 6 kg/m^2^ and handgrip strength < 16 kg.

#### Exercise tolerance

Exercise tolerance was assessed by a 6-min walk, performed according to American Thoracic Society standards [30].

### Pulmonary function

Spirometry testing was conducted by an experienced pulmonary technician using a portable spirometer (EasyOnePC, Ndd Medical Technologies Inc., Zürich, Switzerland) according to the criteria of the American Thoracic Society/European Respiratory Society standards [31]. The spirometry variables analyzed were FEV_1_, and FVC after using a bronchodilator. After 15 min resting, a maximum forced inhalation and a powerful forced expiration were performed by the participant wearing a nose clip. The reference values used for spirometry were obtained in Mexican–American individuals [32].

### Statistical analysis

Analyses were performed using the commercially available package STATA version 14 (Stata Corp., College Station, TX, U.S.A.). The Shapiro–Wilk test was used to test the normality of continuous variables. Normal continuous variables were expressed as mean and standard deviation, while non-normal variables were expressed as median and percentiles 25–75. A comparison among study groups was analyzed with a chi-square test for categorical variables and unpaired Student's t-test or Wilcoxon tests for continuous variables.

Linear regression analysis was performed to examine the association between the dependent variable: pulmonary function (predicted FEV_1_% and predicted FVC %) and independent variables: body composition compartments, handgrip strength, exercise tolerance, and sarcopenia. The models were adjusted for sex, height, and age. A *p-value* < 0.05 was considered statistically significant.

## Results

One hundred eighty-five patients with COPD were evaluated, of whom 78 (42%) had sarcopenia. The mean population age was 72.20 ± 8.39 years; 55.14% were men, 50.27% had systemic hypertension, 29.19% were obese, and 51.80% were in heart failure.

 COPD patients with sarcopenia were older, with a lower prevalence of obesity, low FEV_1_ (L), FVC (L), BMI, FM, FFM, handgrip strength, middle-upper arm circumference, SMMI, ASMMI, abdominal obesity, PhA, and exercise tolerance compared to those COPD patients without sarcopenia (Table 1).

**Table 1:** Clinical and body composition characteristic in sarcopenia subjects

	Total population n = 185	Sarcopenia n = 78	No sarcopenia n = 107	p-value
Age, years	72.20 ± 8.39	74.56 ± 7.60	70.48 ± 8.55	0.001
Male, n (%)	102 (55.14)	45 (57.69)	57 (53.27)	0.550
Diabetes, n (%)	44 (23.78)	17 (21.79)	27 (25.23)	0.587
Hypertension, n (%)	93 (50.27)	38 (48.72)	55 (51.40)	0.718
Obesity, n (%)	54(29.19)	6 (7.69)	48 (44.86)	< 0.001
Heart failure, n (%)	72 (51.80)	34 (58.62)	38 (46.91)	0.173
FEV_1_, (%)	55.64 ± 22.64	53.30 ± 24.87	57.34 ± 20.82	0.230
FEV_1_, (L)	1.22 ± 0.58	1.09 ± 0.56	1.31 ± 0.57	0.009
FVC, (%)	74.92 ± 19.40	73.15 ± 21.06	76.21 ± 18.08	0.290
FVC (L)	2.29 ± 0.81	2.13 ± 0.78	2.41 ± 0.81	0.020
FEV_1_/FVC	0.53 ± 0.13	0.50 ± 0.15	0.54 ± 0.12	0.081
GOLD stage, n (%)				
1–2	105 (56.76)	43 (55.13)	62 (57.94)	
3–4	80 (43.24)	35 (44.87)	45 (42.06)	0.703
CAT score	12 [7–18]	12 [9–20]	11 [6–16]	0.080
Group, n (%)				
A–B	94 (53.41)	35 (47.95)	59 (57.28)	0.221
C–D	82 (46.59)	38 (52.05)	44 (42.72)	
Hospitalization previous year, n (%)	81 (46.82)	39 (54.17)	42 (41.58)	0.102
Tobacco index, pack-year	40 [25–60]	40 [20–58]	43 [30–61.5]	0.164
Biomass index, h/yr	213 [77.5–360]	240 [80–385]	180 [51–360]	0.584
Inhaled bronchodilators, n (%)	144 (78.26)	64 (82.05)	80 (75.47)	0.285
Inhaled corticosteroids, n (%)	117 (66.30)	52 (66.67)	70 (66.04)	0.929
Height, cm^2^	158.00 ± 11.39	156.31 ± 12.35	159.23 ± 10.52	0.085
Body Mass Index, kg/m^2^	27.26 ± 5.70	24.05 ± 4.50	29.61 ± 5.34	< 0.001
Fat Mass, kg	30.39 ± 10.03	27.51 ± 8.22	32.44 ± 10.71	< 0.001
Fat Free Mass, kg	37.79 ± 12.05	30.92 ± 8.91	42.70 ± 11.63	< 0.001
Handgrip strength, kg	23.72 ± 8.47	20.37 ± 7.76	26.22 ± 8.15	< 0.001
Middle-upper arm circumference, cm	28.22 ± 4.25	25.92 ± 3.84	29.93 ± 3.71	< 0.001
SMMI, kg/m^2^	8.31 ± 1.85	7.43 ± 1.44	8.94 ± 1.86	< 0.001
ASMMI, kg/m^2^	6.79 ± 1.14	6.03 ± 0.74	7.35 ± 1.07	< 0.001
Abdominal obesity, n (%)	140 (76.92)	45 (58.44)	95 (90.48)	< 0.001
Phase angle, °	4.99 ± 0.88	4.57 ± 0.54	5.30 ± 0.95	< 0.001
Exercise tolerance, m	324.34 ± 131.97	267.24 ± 128.12	388.58 ± 104.88	< 0.001

*FEV*_*1*_, Forced Expiratory Volume in the first second; *FVC* Forced Vital Capacity; *GOLD stage* Global Initiative for Chronic Obstructive Lung Disease stage; *CAT* COPD Assessment Test; *SMMI* Skeletal muscle mass index; *ASMMI* Appendicular Skeletal muscle mass index

Table 2 shows the linear regression results adjusted for age, sex, and height, in which the variables associated with FEV_1_ and FVC are presented. BMI (β:0.670, 95% CI; 0.181–1.159), FFM (β:0.648, 95% CI;0.217–1.079), middle-upper arm circumference, SMMI, ASMMI, PhA, and exercise tolerance were positively associated with FEV_1_. Sarcopenia, however, had a negative association with FEV_1_. As regards the FVC, PhA and exercise tolerance showed positive associations (β:0.064, 95% CI; 0.036–0.093 for exercise tolerance).

**Table 2:** Association between body composition, sarcopenia and pulmonary function

	FEV_1_ (%)			FVC (%)		
	β	CI 95%	*p*	β	CI 95%	*p*
Body Mass Index, kg/m^2^	0.670	0.181 to 1.1590	0.007	0.061	− 0.377 to 0.500	0.783
Fat Mass, kg	0.027	− 0.294 to 0.349	0.868	− 0.129	− 0.421 to 0.162	0.383
Fat Free Mass, kg	0.648	0.217 to 1.079	0.003	0.233	− 0.157 to 0.624	0.241
Handgrip strength, kg	0.037	− 0.566 to 0.641	0.902	0.448	− 0.076 to 0.974	0.094
Upper extremity weakness	3.078	− 3.959 to 10.116	0.389	− 3.687	− 10.196 to 2.821	0.265
Middle-upper arm circumference, cm	0.884	0.205 to 1.564	0.011	0.227	− 0.379 to 0.833	0.461
SMMI, kg/m^2^	2.876	0.534 to 5.218	0.016	0.894	− 1.212 to 3.001	0.403
ASMMI, kg/m^2^	4.896	1.982 to 7.810	0.001	1.388	− 1.228 to 4.00	0.297
Abdominal obesity	5.902	− 1.059 to 12.864	0.096	− 0.175	− 6.350 to 5.999	0.955
Phase angle, °	5.745	2.596 to 8.895	< 0.001	3.871	1.077 to 6.665	0.007
Sarcopenia	− 6.992	− 13.722 to − 0.261	0.042	− 5.090	− 11.007 to 0.826	0.091
Exercise tolerance, m	0.057	0.024 to 0.090	0.001	0.064	0.036 to 0.093	< 0.001

Linear regression model adjusted by age, sex and height. *SMMI* Skeletal muscle mass index; *ASMMI* Appendicular Skeletal muscle mass index

## Discussion

The main finding in our study was that FEV_1_ had a positive correlation with SMMI, ASMMI, BMI, FFM, PhA, middle-upper arm circumference, and exercise tolerance, while sarcopenia had a negative impact. As far as FVC was concerned, PhA and exercise tolerance showed positive associations.

Muscle mass is the largest tissue in the human body [33]. It can also be an independent prognostic factor for daily disability, mobility, and mortality in COPD patients [10, 12, 34]. Our study showed positive associations between FEV_1_, and ASMMI (β: 4.896, 95% CI; 1.982 to 7.810, *p* = 0.001) and SMMI (β: 2.876, 95% CI; 0.534 to 5.218, *p* = 0.016). That is, for every 1 kg/m^2^ of ASMMI that increased, there was an increase of 4.89% in FEV_1_, and for every 1 k/m^2^ of SMMI, FEV_1_ increased 2.87%. Previous studies have also reported that subjects with low muscle mass have worse pulmonary function. A study performed by Jeon et al. in elderly Koreans found that subjects with low FEV_1_ were at higher risk for decreased muscle mass [15]. Also, a study performed by Park et al. in subjects without pulmonary disease showed that lower muscle mass was associated with a higher risk of FEV_1_ < 80% (OR: 2.97, CI 95% 2.74 to 3.17) and FVC < 80% (OR: 2.64, CI 95%; 2.43 to 2.83) [16].

On the other hand, in the present study, a higher BMI was also positively associated with FEV_1_. Currently, a controversy exists about the impact of obesity evaluated by BMI on pulmonary function and prognostic patients, which has been called the paradox of obesity [35, 36]. There is evidence that a higher BMI improves pulmonary function [36, 37]. A meta-analysis performed on COPD patients from clinical trials observed that overweight-obesity and morbid obesity subjects evaluated by BMI had minor decreases in FEV_1_ per year compared to normal weight and underweight subjects [37]. However, evidence shows the deleterious effects of obesity on pulmonary function [38–40]. While a study by Ochs-Balcom et al. found a negative association between BMI > 25 kg/m^2^ with both FEV_1_ and FVC, a study by Peralta et al. reported that increased weight in overweight and obese subjects was associated with a reduction only in FVC. However, no association was observed with the FEV_1_/FVC ratio [39]. Similar results have been reported in other studies [41, 42].

Nevertheless, it is essential to bear in mind that BMI is the quotient of body weight in kg and height in m^2^, but BMI cannot distinguish the two most significant components of body composition: FM and FFM. Our study observed that FFM positively impacted FEV_1_, while no such association was observed with fat mass when evaluating these two components. These results suggest that the FFM, specifically SMMI or ASMMI, plays an essential role in pulmonary function. Won et al. likewise observed that SMMI was associated with FEV_1_ and FVC [43]. Apart from FFM, SMMI and ASMMI correlate with prognosis [10–12].

In this study PhA proved to be a predictor for FEV_1_ (β: 5.745, CI 95%; 2.596 to 8.895, *p* < 0.001) and FVC (β: 3.871, CI 95%; 1.077 to 6.665, *p* = 0.007) adjusted for age, sex and height. That is, for every 1 degree that PhA increases, there is an increment of 5.74% for FEV_1_ and 3.87% for FVC.

A lower PhA has been associated with reduced FEV_1_, muscle strength, exercise tolerance, diminished quality of life, prolonged hospitalization, and exacerbations [13], as well as being an independent predictor of death (HR: 0.53, CI 95%; 0.36–0.77 *p* < 0.001) in COPD subjects [14].

It is noteworthy that muscle strength is a significant predictor of mortality [12]. Although an association between diminished handgrip strength and pulmonary function was not observed in the present study, it was found in other studies. Liu et al. reported a positive correlation between the strength of upper limb muscles and inspiratory and expiratory respiratory muscles and the FEV_1_/FVC ratio [5]. Likewise, subjects hospitalized because of exacerbations of COPD showed a positive association between handgrip strength and effective peak inspiratory flow rate determined by the inspiratory muscle force [44].

As regards sarcopenia, the prevalence in COPD patients is 22% [21], representing an increase according to the GOLD [20, 22]. The present study demonstrated that sarcopenia was an independent predictor for FEV_1_ since subjects had 6.992% lower FEV_1_ than those without sarcopenia (β: − 6.992, 95% CI; − 13.722 to − 0.261, *p* < 0.042) adjusted for age, sex, and height. Different studies have shown that sarcopenia, muscle depletion, and low muscle strength are associated with deteriorating lung function, higher inflammatory biomarkers, poor quality of life, and worse prognosis [10, 19, 20, 45].

When adipose tissue distribution is examined in the context of obesity, abdominal obesity plays an important role in pulmonary function. A study in subjects over 50 years of age showed that those with abdominal obesity had decreased pulmonary function. Moreover, pulmonary function was even lower in those with generalized obesity [42]. In a meta-analysis Wehrmeister et al. found similar results. They concluded that an inverse association exists between pulmonary function and abdominal circumference [41], which is a good indicator of abdominal obesity. This could be explained by the fact that obesity causes dysfunction in small airways and limitation of expiratory flow as well as mechanical respiratory changes, restricted movement of the thoracic cage, decreased strength of the respiratory muscles, gas exchange, and reduced tolerance to exercise due to excess adipose tissue around the rib cage and abdomen [46]. The present study did not establish an association between abdominal obesity and pulmonary function, but this may be explained by the population's higher prevalence of sarcopenia.

In addition, obesity, especially central obesity, is associated with an increase in inflammatory factors such as interleukin 6 and tumor necrosis α [47], which negatively impact pulmonary function and increase morbidity and mortality [48]. That is why it is essential to distinguish among body composition compartments–FM, FFM, ASMMI, SMMI, and PhA which have different impacts on pulmonary function and prognosis, both in healthy subjects and in COPD patients.

The clinical picture is further complicated by comorbidities such as hypertension, diabetes, and heart failure, which are frequent in COPD [49]. These pathologies are independent risk factors for sarcopenia [49, 50]. The presence of one or more comorbidities leads to more significant loss of skeletal muscle mass, physical performance, and worse prognosis [51]. This low skeletal muscle mass had a negative impact on pulmonary function [15, 16].

### Limitations and strengths

This study has the inherent limitations of a cross-sectional study. Another limitation is the small sample size. However, among the strengths, this is the first study in COPD patients that evaluates the association of different compartments of body composition on pulmonary function using a multivariate prediction model adjusted for confounding variables.

## Conclusions

The components of body composition, especially PhA, SMMI, ASMMI, and sarcopenia, have significant impacts on pulmonary function. Early detection of disturbances in these indexes and muscle wasting enables the early application of such therapeutic strategies as physical and pulmonary rehabilitation, and nutritional treatment may improve pulmonary function in COPD patients.

## Data Availability

The datasets generated and/or analyzed during the current study are not publicly available due to the fact that individual privacy could be compromised but are available from the corresponding author on reasonable request.

## Abbreviations

ASMMI: Appendicular skeletal muscle mass index
BIA: Bioelectrical impedance analysis
BMI: Body Mass Index
COPD: Chronic Obstructive Pulmonary Disease
FM: Fat mass
FEV_1_: Forced expiratory volume in first second
FEV_1_/FVC: Forced expiratory volume in first second/Forced vital capacity
FFM: Fat-free mass
FVC: Forced vital capacity
GOLD: Global Initiative for Chronic Obstructive Lung Disease
PhA: Phase angle
SMMI: Skeletal muscle mass index
